# Quantifying Epistemic Uncertainty in Multimodal Long-Tailed Classification: A Belief Entropy-Based Evidential Fusion Framework

**DOI:** 10.3390/e28030343

**Published:** 2026-03-19

**Authors:** Guorui Zhu

**Affiliations:** 1 School of Business Administration, Southwestern University of Finance and Economics, Chengdu 611130, China; 1221201z5007@smail.swufe.edu.cn; 2 Institute of Big Data, Southwestern University of Finance and Economics, Chengdu 611130, China

**Keywords:** belief entropy, long-tailed classification, Dempster-Shafer Theory, uncertainty quantification, multimodal information fusion

## Abstract

Deep multimodal learning has excelled in tasks involving vision, language, and audio modalities. Nevertheless, their performance on tail classes exhibits significant degradation under the long-tailed distributions common in real-world data, meanwhile related fusion schemes often provide only limited treatment of modality-specific uncertainty and rarely incorporate explicit mechanisms for class-level fairness. To address these information discrepancies, we present a framework that integrates evidential reasoning with deep learning–Uncertainty-Quantified Multimodal Learning for Long-Tailed Classification (UMuLT). The framework includes: (i) an uncertainty-gated evidential fusion module that adaptively down-weights unreliable modalities; (ii) an exponential moving average (EMA) fairness regularizer that dynamically amplifies tail-class gradients; and (iii) a cross-modal consistency regularizer optimized in two stages: tail specialization with lightweight adapters on tail-class data to obtain a balanced initialization, followed by end-to-end fine-tuning. The effectiveness and practicality of our method are verified on three long-tailed benchmarks for multimodal classification. Experiments show consistent gains over strong baselines in overall metrics, calibration, and tail subset performance. Statistical significance tests confirm the superiority of the proposed framework.

## 1. Introduction

The fundamental premise of multimodal learning is to enhance decision reliability by synthesizing heterogeneous signals—such as text, audio, and video—thereby mimicking human perception in complex environments [1]. While this paradigm has demonstrated robustness in tasks ranging from conversational emotion recognition [2] to 3D detection [3], the integration process is rarely a simple additive gain. Instead, it frequently introduces “cross-modal uncertainty” stemming from sensor noise and inherent discrepancies between modalities, which can severely constrain generalization [4]. This reliability gap widens significantly in real-world scenarios governed by power-law distributions [5]. Here, the system faces a “double jeopardy”: tail classes suffer from epistemic uncertainty due to label scarcity, compounded by aleatoric uncertainty from imperfect sensor data. Such a high-entropy state creates a critical vulnerability, directly undermining the trustworthiness of expert systems in safety-critical deployments.

In practice, multimodal heterogeneity and long-tailed distributions are not isolated challenges but inextricably linked phenomena. Rare categories are naturally prone to data degradation or modal absence, leading to a destructive compounding of uncertainty and class imbalance [6]. The cost of failure in these scenarios is disproportionately high, particularly in equity-sensitive applications where misclassifying rare events can have severe consequences [7]. Given that stochastic channel degradation and annotation costs often preclude the curation of perfectly balanced datasets, a robust system must possess the cognitive flexibility to dynamically allocate trust. Much like human evidential reasoning [8,9], the model must weigh conflicting evidence under uncertainty, necessitating a unified theoretical lens that simultaneously addresses modality discrepancy and class skew.

Despite these needs, a methodological schism persists in the current literature. Multimodal fusion and long-tailed recognition are typically treated as orthogonal optimization problems. Conventional fusion strategies [10,11,12] focus on maximizing information interaction but often ignore the underlying class distribution, causing tail classes to be drowned out during evidence accumulation. Conversely, standard long-tailed techniques, whether through reweighting [13] or feature generation [14,15], implicitly assume that input features are reliable and clean. Even recent advances in vision-language adaptation [16,17] and uncertainty quantification [18] fail to explicitly couple reliability estimation with class re-balancing in a multimodal context [19]. Consequently, a unified framework capable of navigating modality-wise uncertainty while ensuring tail-class fairness remains largely absent.

To resolve this dichotomy, we introduce the Uncertainty-Quantified Multimodal Learning for Long-Tailed Classification (UMuLT) framework. The central concept of UMuLT is the explicit decoupling of aleatoric uncertainty (inherent data noise) from epistemic uncertainty (model ignorance due to scarcity). Moving beyond traditional point-based probabilities, UMuLT leverages Dempster-Shafer Theory to model predictions as belief masses. A pivotal innovation in our approach is the deployment of Belief Entropy as an information-theoretic probe. Unlike Shannon entropy, which conflates “ignorance” (lack of data) with “conflict” (disagreeing modalities), Belief Entropy explicitly distinguishes between them. This distinction is crucial: it allows the system to rigorously filter out noisy signals while actively identifying under-represented classes, preventing noise from corrupting decisions on rare classes.

Our optimization strategy is decoupled into quality assessment and quantity re-balancing. We first need a representation that can tell apart noisy modalities from ignorant ones; Dempster-Shafer belief masses provide exactly this. We then need a fusion strategy that acts on this distinction, suppressing noise while keeping useful signals aligned; the entropy gate and consistency regularizer serve this role. Finally, even with cleaner fusion, training on imbalanced data still biases the model toward head classes, so the EMA fairness regularizer and two-stage protocol handle the remaining distributional skew.

Our main contributions are as follows:

1. We identify a compounding effect between modality-level noise and class-level scarcity that existing multimodal and long-tailed methods address only in isolation. In real-world data, rare classes tend to suffer from both insufficient samples and degraded modality quality simultaneously, yet current fusion strategies assume clean inputs while current re-balancing strategies assume reliable features. To tackle both issues within a single framework, we ground UMuLT in Dempster-Shafer Theory, which represents each modality’s output as belief masses rather than point probabilities, enabling an explicit separation of aleatoric uncertainty from epistemic uncertainty at the modality level.
2. We propose a fusion mechanism gated by Belief Entropy, which serves as a more informative alternative to Shannon entropy for multimodal settings. Shannon entropy conflates two fundamentally different situations: high entropy caused by conflicting modality evidence and high entropy caused by insufficient evidence. Belief Entropy distinguishes between them, allowing the gate to suppress genuinely noisy modalities while retaining uncertain but informative ones. We further pair this gate with a cross-modal consistency regularizer that enforces semantic alignment across modalities after gating, so that the surviving evidence is not only individually reliable but also mutually coherent.
3. Improved fusion alone does not resolve the class imbalance embedded in the training distribution. To complement the upstream fusion mechanism, we introduce a fairness regularizer based on exponential moving average of class-wise losses. Unlike static re-weighting schemes derived from class frequencies, this regularizer tracks each class’s actual learning progress throughout training and amplifies the gradient signal for classes that are persistently under-fitting. Combined with a two-stage training protocol where lightweight adapters are first tuned on tail-class data before full model fine-tuning, this ensures that the cleaner fused representations actually translate into better tail-class recognition.

The remainder of this paper is organized as follows. We first review previous works related to multimodal fusion and long-tailed recognition strategies in Section 2. Section 3 explains the details of our method. Section 4 shows the experimental setup and results. Section 5 discusses the results and their implications. Finally, Section 6 concludes the paper and outlines directions for future work.

## 2. Related Work

### 2.1. Multimodal Fusion and Evidential Reasoning Methods

In the past, people used simple methods like early fusion or late fusion. Now, deep learning is the main approach. Many recent works use attention mechanisms [10,20] or Transformers [21] to combine different modalities. Some researchers also use graph neural networks [11] or add fairness constraints [22]. Although these methods are good, they usually output a deterministic probability. Conventional approaches typically rely on the softmax function, which yields point estimates for class probabilities. However, this formulation collapses the total uncertainty, failing to decouple aleatoric uncertainty (data noise) from epistemic uncertainty (model ignorance). Consequently, in scenarios involving missing or noisy modalities, these models frequently exhibit detrimental overconfidence [19].

Evidential Deep Learning (EDL) is a good way to solve this. It is based on Dempster-Shafer Theory (DST) [9]. DST assigns “belief masses” to classes, which allows the model to represent ignorance explicitly. Unlike standard probability theory which distributes total mass over individual classes, DST assigns mass to the power set of classes. This allows the framework to model lack of evidence separately from conflicting evidence, providing a mathematical basis for robust decision-making under open-world conditions. Some works have applied DST to multimodal fusion [23]. Recent advancements have significantly enhanced the reliability of such systems in complex environments. Xu et al. [24] notably addressed the issue of conflicting views by modeling view-specific reliability, an approach further extended to safety-critical scenarios for explicit uncertainty quantification [25]. Beyond conflict resolution, researchers have focused on mitigating data imperfections, developing robust mechanisms to handle noisy supervision [26] and effectively recover missing information in incomplete settings [27,28]. These efforts have evolved into more sophisticated strategies, such as dynamic evidence decoupling to isolate valid signals from noise [29], and fairness-aware mechanisms designed to correct biased evidence allocation through adaptive priors [30].

### 2.2. Long-Tailed Learning Methods

Long-tailed recognition is a well-studied problem. Common methods include re-sampling [31] and re-weighting [13,32]. These methods give more importance to tail classes during training. Recently, some papers propose two-stage training [14,31] or using multiple experts [5,33]. Transfer learning from large models is also popular [16,17].

However, these methods have a limitation: they assume the input image or text is always good. They focus on fixing the label imbalance but forget about feature quality. In multimodal tasks, tail samples often have missing or bad modalities [19]. If we just force the model to learn these bad samples without checking quality, it might learn noise [3].

### 2.3. Uncertainty Quantification and Belief Entropy Methods

Quantifying model uncertainty is paramount for ensuring the safety and reliability of deep learning systems. Uncertainty is typically categorized into two distinct types: aleatoric uncertainty, arising from inherent data noise, and epistemic uncertainty, stemming from model ignorance [18]. While traditional approaches, such as Bayesian Neural Networks, offer rigorous uncertainty estimation, they are often hindered by high computational costs. Consequently, Evidential Deep Learning (EDL) has emerged as a prominent alternative, favored for its efficiency and deterministic nature [9]. Within this framework, Belief Entropy serves as a critical metric, effectively measuring both evidence conflict and distributional ignorance [34,35].

Despite the availability of these mathematical tools, no existing method simultaneously couples belief entropy-guided fusion with long-tailed re-balancing in a multimodal setting. Existing uncertainty-aware methods typically operate in unimodal settings or focus on balanced datasets, failing to account for the specific information dynamics where tail classes suffer from both label scarcity and modality corruption. This work bridges this gap by utilizing belief entropy not merely as a passive metric but as an active information-theoretic probe to guide the fusion process and rectify the optimization bias in long-tailed learning.

## 3. Methodology

**Figure 1:**
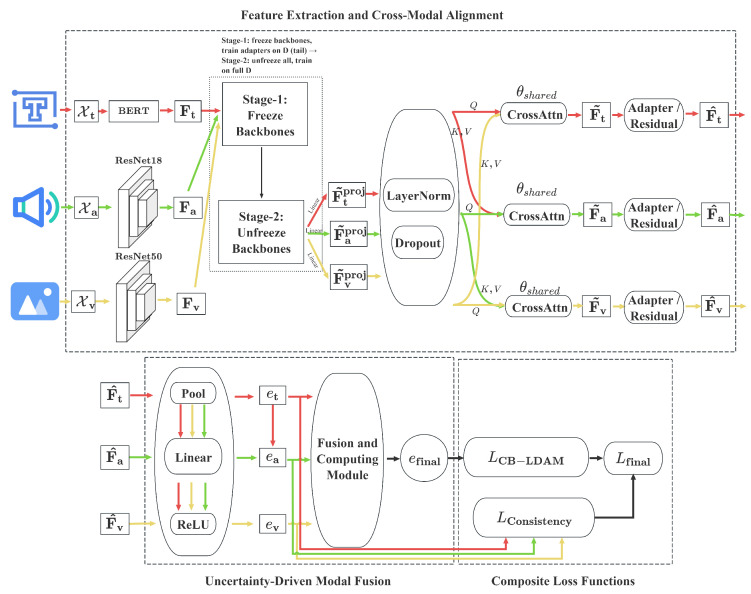
Overall framework of the UMuLT.

In this section, we introduce UMuLT, a unified classification framework for multimodal data with long-tailed distribution, as shown in Figure 1 and Algorithm 1. The framework consists of three key modules: Feature Extraction and Alignment, Evidence Computation and Fusion, and Loss Computation and Parameter Update. These three modules constitute the core implementation of our approach, translating the theoretical distinction between aleatoric and epistemic uncertainty into a differentiable deep learning pipeline. Together, these modules address challenges posed by modality discrepancies and head–tail class imbalance.

We address class overlap and fuzzy boundaries in long-tailed data by enforcing cross-modal consistency that aligns modality specific evidence for the same instance and by letting fusion favor signals that agree, which sharpens decision margins for rare classes. EMA fairness increases tail evidence, consistency mechanism reduces conflict and stabilizes gating, and gated fusion filters unreliable modalities and sharpens the signal, together forming a positive feedback loop that improves accuracy and calibration in multimodal long-tail classification. The following subsections detail the formulations and implementation aspects of each stage, covering the network architecture, uncertainty estimation, loss function design, and overall training procedure.

Computational Cost and Deployment Efficiency. While UMuLT introduces additional computational overhead during training to explicitly model cross-modal alignment and uncertainty, this is a justified investment for building a reliable decision support system. Without evidential reasoning, rare classes tend to have overlapping boundaries and unstable calibration. To ensure practical deployment feasibility, we implement inference-time mechanisms such as text-only early exit and on-demand skipping of the alignment block. These strategies significantly reduce the average latency by allocating computational resources only to hard instances near decision boundaries. Consequently, the framework achieves a balanced trade-off between reliability and deployment efficiency, meeting the requirements of real-time industrial applications.
**Algorithm 1** UMuLT Model Framework**Require:** 
Training set D, modalities M={t,a,v}, classes *K***Ensure:** 
Trained parameters θ  1:Initialize EMA loss tracker ${\dot{L}}_{k}\leftarrow 0$ for all k∈{1,…,K}  2:***Stage 1: Tail Specialization***  3:Freeze backbones; train adapters on $D_{\mathrm{tail}}$  4:***Stage 2: Global Fine-Tuning***  5:Unfreeze all parameters  6:**for** each epoch *p* **do**  7:    **for** each mini-batch B⊂D **do**  8:       ***Step 1: Feature Extraction & Alignment***  9:       **for** m∈M **do**10:           ${\hat{F}}_{m}\leftarrow \mathrm{Adapter}\left(\mathrm{CrossAttn}\left({\mathrm{Encoder}}_{m}\left(X_{m}\right)\right)\right)$11:       **end for**12:       ***Step 2: Evidence & Uncertainty Computation***13:       **for** m∈M **do**14:           $e_{m}\leftarrow \mathrm{ReLU}\left(\mathrm{Linear}\left(\mathrm{Pool}\left({\hat{F}}_{m}\right)\right)\right)$15:           $S_{m}\leftarrow \sum_{k}e_{k,m}+K$;    $u_{m}\leftarrow K/S_{m}$16:           $b_{k,m}\leftarrow e_{k,m}/S_{m}$;    ${\tilde{b}}_{k,m}\leftarrow b_{k,m}/\left(1-u_{m}\right)$17:       **end for**18:       ***Step 3: Uncertainty-Gated Fusion***19:       $w_{t}\leftarrow 1-u_{t}$ {Text as anchor}20:       $C_{a}\leftarrow 1-\langle {\tilde{b}}_{a},{\tilde{b}}_{t}\rangle$;    $w_{a}\leftarrow \left(1-u_{a}\right)\left(1-C_{a}\right)$21:       $C_{v}\leftarrow 1-\langle {\tilde{b}}_{v},{\tilde{b}}_{a}\rangle$;    $w_{v}\leftarrow \left(1-u_{v}\right)\left(1-C_{v}\right)$22:       $\tilde{a}\leftarrow \mathrm{softmax}\left(\left[w_{t},w_{a},w_{v}\right]/\eta \right)$23:       **for** m∈M **do**24:           **if** $u_{m}>\tau_{\mathrm{unc}}$ **then**25:               ${\tilde{a}}_{m}\leftarrow {\tilde{a}}_{m}\cdot \max\left(0.01,\;1-\kappa \left(u_{m}-\tau_{\mathrm{unc}}\right)\right)$26:           **end if**27:           $a_{m}\leftarrow {\tilde{a}}_{m}\exp\left(-u_{m}\right)/\sum_{n}{\tilde{a}}_{n}\exp\left(-u_{n}\right)$28:       **end for**29:       $e_{\mathrm{final}}\leftarrow \sum_{m}a_{m}\;e_{m}$30:       ***Step 4: Loss Computation***31:       **for** sample i∈B **do**32:           $\Delta_{y_{i}}\leftarrow \varpi /n_{y_{i}}^{1/4}$;    $\gamma_{y_{i}}\leftarrow \left(1-\beta \right)/\left(1-\beta^{n_{y_{i}}}\right)$33:           $L_{\mathrm{cls},i}\leftarrow -\gamma_{y_{i}}\log\mathrm{softmax}{\left(e_{\mathrm{final}}-\Delta_{y_{i}}1_{y_{i}}\right)}_{y_{i}}$34:       **end for**35:       **for** class *k* **do**36:           ${\dot{L}}_{k}\leftarrow \psi {\dot{L}}_{k}+\left(1-\psi \right)\cdot \mathrm{mean}\left(L_{\mathrm{cls}}\;\mathrm{for}\;\mathrm{class}\;k\right)$37:       **end for**38:       $\lambda_{k}\leftarrow \mathrm{softmax}{\left(\lambda_{\mathrm{fair}}\left(\dot{L}-\bar{L}\right)\right)}_{k}$ for all *k*39:       $L_{\mathrm{cons}}\leftarrow \frac{1}{\left|B\right|}\sum_{i}\sum_{m<n}{∥e_{m,i}-e_{n,i}∥}^{2}$40:       $L\leftarrow \sum_{i}\lambda_{y_{i}}L_{\mathrm{cls},i}+\delta L_{\mathrm{cons}}$41:       $\theta \leftarrow \theta -\eta_{l}\nabla_{\theta }L$42:   **end for**43:**end for**44:**return** θ

### 3.1. Feature Extraction and Cross-Modal Alignment

Let $X_{t}$, $X_{a}$, and $X_{v}$ denote the raw text, audio, and visual inputs, respectively. The textual input $X_{t}$ is encoded using a BERT-base encoder with a maximum of $L_{t}$ tokens [36]; we retain token-level representations $F_{t}\in R^{L_{t}\times d_{t}}$. For audio, 16 kHz audio is converted to Mel-frequency cepstral coefficients (MFCCs) and processed by a 1D ResNet-18; we retain frame-level features $F_{a}\in R^{L_{a}\times d_{a}}$. For video, frames are uniformly sampled and passed through an ImageNet-pretrained ResNet-50; we retain per-frame (or patch) features $F_{v}\in R^{L_{v}\times d_{v}}$ for alignment, while temporal averaging is reserved for ablations.

Before projection, each modality feature $F_{m}\in R^{L_{m}\times d_{m}}$ resides in its own dimensional space. After a linear projection into the shared space, we obtain $F_{m}^{\mathrm{proj}}\in R^{L_{m}\times d}$, followed by LayerNorm and dropout.

(1) $$\mathrm{Adapter}\left({\tilde{F}}_{m}\right)={\tilde{F}}_{m}\;+\;W_{m}^{\mathrm{up}}\;\phi \;\left(W_{m}^{\mathrm{down}}\;{\tilde{F}}_{m}+b_{m}^{\mathrm{down}}\right)+b_{m}^{\mathrm{up}},$$

where $W_{m}^{\mathrm{down}}\;\in \;R^{d\times r}$, $W_{m}^{\mathrm{up}}\;\in \;R^{r\times d}$, ϕ is ReLU, and r < d is the bottleneck dimension. The output is denoted by ${\hat{F}}_{m}$. $W_{m}^{\mathrm{down}}\in R^{d\times r}$ and $W_{m}^{\mathrm{up}}\in R^{r\times d}$ project to and from the bottleneck *r*, with biases $b_{m}^{\mathrm{down}}\in R^{r}$ and $b_{m}^{\mathrm{up}}\in R^{d}$.

Specifically, we retain modality-specific backbones (BERT/ResNet) to extract unimodal features, and then introduce a Transformer-based cross-modal alignment encoder [37] that operates on the projected features from all modalities. This alignment encoder is shared across modalities (parameter tying) and learns cross-modal dependencies, thereby reducing redundancy and improving computational efficiency without replacing the unimodal backbones.

To align the different modalities, we introduce a shared Cross-Attn module. In the original design, each modality performs independent Cross-Attn operations for alignment, but to simplify the structure and improve efficiency, we reuse a single set of Cross-Attn parameters and apply the same block once per modality (one pass per modality as query). This shared-parameter attention allows information exchange between modalities in a unified manner, reducing parameter count and minimizing computational overhead.

Each modality-specific representation $F_{m}$ is first projected into a shared representation space of dimensionality *d* via a linear transformation, resulting in ${\tilde{F}}_{m}$. The shared Cross-Attn mechanism then aligns the modalities as follows:

(2) $${\tilde{F}}_{t}=\mathrm{SharedCrossAttn}\left(\mathrm{Query}=F_{t}^{\mathrm{proj}},\;\mathrm{Key}=\mathrm{Value}=\mathrm{Concat}\left[F_{a}^{\mathrm{proj}};\;F_{v}^{\mathrm{proj}}\right]\right),$$

(3) $${\tilde{F}}_{a}=\mathrm{SharedCrossAttn}\left(\mathrm{Query}=F_{a}^{\mathrm{proj}},\;\mathrm{Key}=\mathrm{Value}=\mathrm{Concat}\left[F_{t}^{\mathrm{proj}};\;F_{v}^{\mathrm{proj}}\right]\right),$$

(4) $${\tilde{F}}_{v}=\mathrm{SharedCrossAttn}\left(\mathrm{Query}=F_{v}^{\mathrm{proj}},\;\mathrm{Key}=\mathrm{Value}=\mathrm{Concat}\left[F_{t}^{\mathrm{proj}};\;F_{a}^{\mathrm{proj}}\right]\right).$$

At inference time, we adopt an on-demand skipping strategy to reduce unnecessary computations. If all available modalities are confident and mutually consistent, we skip the shared Cross-Attn operation, thus reducing the computational cost. Confidence and agreement are computed from per-modality evidence heads applied before the shared Cross-Attn. During training, however, we always perform the shared Cross-Attn operation to preserve alignment capacity and ensure stable gradients. Instead, the fusion stage uses $e_{m}$ computed from the aligned-and-adapted features ${\hat{F}}_{m}$.

This approach simplifies the architecture by avoiding the need for separate attention mechanisms for each modality. The shared Cross-Attn mechanism processes all modalities through a unified attention mechanism, reducing the number of operations and memory usage while maintaining efficient cross-modal interaction.

To efficiently fuse multimodal features, we apply the lightweight bottleneck adapter defined above to each aligned representation:

(5) $${\hat{F}}_{m}\;=\;\mathrm{Adapter}\left({\tilde{F}}_{m}\right),$$

where the adapter uses a two-layer bottleneck d→r→d with parameters $W_{m}^{\mathrm{down}}\in R^{d\times r}$ and $W_{m}^{\mathrm{up}}\in R^{r\times d}$ (see the adapter block above). This preserves the residual shape and enables parameter-efficient modulation in the shared space *d*.

Training is performed in two stages:

**Stage 1—Tail Specialization.** The backbone networks are frozen, and only the adapters and classification heads are trained on the tail samples $\left(x_{i},y_{i}\right)|y_{i}\in Dataset_{\mathrm{tail}}$, enabling the adapters to specialize in tail-class cues. This stage isolates tail-class representations before joint optimization.

**Stage 2—Global Fine-Tuning.** The entire network is unfrozen and jointly optimized on the entire dataset. Crucially, the EMA-based fairness regularizer remains active to protect tail-class gradients. By dynamically amplifying the loss contribution of under-performing classes, the EMA mechanism counteracts the tendency of head-class gradients to dominate the optimization landscape. This explicitly prevents the catastrophic forgetting of tail-specialized features learned in Stage 1, ensuring that the tail-specific knowledge is integrated rather than overwritten during end-to-end fine-tuning.

During alignment, we retain the pre-trained initialization of each modality’s backbone, preserving its domain-specific representational power. The adapter modules and cross-attention mechanisms refine multimodal representations, bridging modality gaps while minimally perturbing the pre-trained features. This design ensures a fair comparison with existing multimodal methods, while allowing the evidential fusion module to fully exploit discriminative information from each modality.

**Figure 2:**
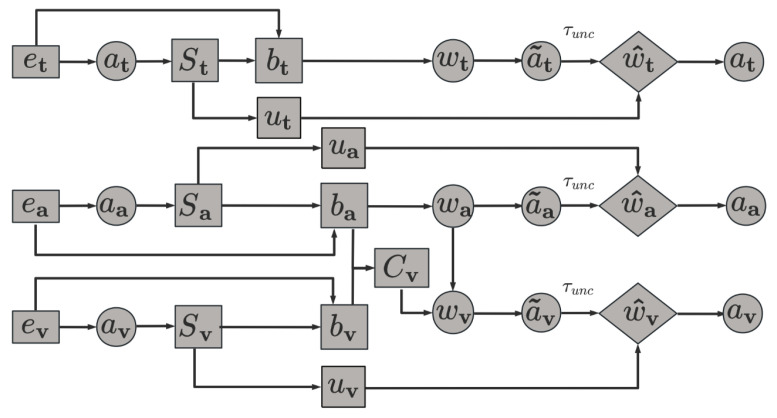
Uncertainty-driven modal fusion.

After obtaining the aligned multimodal features ${\hat{F}}_{m}$, UMuLT performs evidential reasoning to estimate the uncertainty of each modality and adaptively adjusts its contribution to the final prediction. Fusion and normalization are computed only over available modalities (masked sums); missing modalities are excluded from all sums and normalizations rather than treated as zero evidence. Thus the algorithm handles both synchronized and asynchronous inputs. The module framework is shown in Figure 2.

It is worth noting that, if the text modality alone is confident and decisive, meaning its evidential uncertainty is low and the gap between its top-1 and top-2 classes is large, we return the text-only prediction and skip computing audio and video. This early exit reduces latency and FLOPs on the audio/video backbones without affecting accuracy. During training we always compute all modalities to keep gradients consistent. The consistency objective drives modality representations to converge on a common class in ambiguous regions and discourages divergent cues, which reduces boundary ambiguity without extra heuristics.

### 3.2. Evidence Computation and Uncertainty Quantification

The aligned features ${\hat{F}}_{m}$ are pooled along the sequence dimension to produce a global feature vector. This vector is then passed through a linear transformation and a ReLU activation to obtain the evidence vector $e_{m}={\left[e_{1,m},e_{2,m},\ldots ,e_{K,m}\right]}^{⊤}$.

Based on the evidential DST formulation [38], the Dirichlet parameters and the uncertainty mass are as follows:

(6) $$\alpha_{k,m}=e_{k,m}+1,\;S_{m}=\sum_{j=1}^{K}\alpha_{j,m},\;u_{m}=\frac{K}{S_{m}}\in \left(0,1\right],$$

where $u_{m}$ represents the uncertainty mass, the belief mass $b_{k,m}$ is:

(7) $$b_{k,m}=\frac{\alpha_{k,m}-1}{S_{m}}=\frac{e_{k,m}}{S_{m}}\in \left[0,1\right],\;\sum_{k=1}^{K}b_{k,m}=1-u_{m}.$$

For cross-modal similarity or conflict, we use unit-mass beliefs

(8) $${\tilde{b}}_{k,m}\;=\;\left\{\begin{array}{cc} \frac{b_{k,m}}{\;1-u_{m}\;}, & \mathrm{if}\;1-u_{m}\geq \varepsilon ,\\ \frac{1}{K}, & \mathrm{otherwise}, \end{array}\right.\;\varepsilon >0.$$

To strictly quantify the information volume and conflict contained within the evidence, we introduce Belief Entropy as a measure of epistemic uncertainty. Given the mass function *m*, the Belief Entropy $H_{B}\left(m\right)$ is defined as:

(9) $$H_{B}\left(m\right)=-\sum_{A\subseteq \Theta }m\left(A\right)\log_{2}\frac{m(A)}{2^{\left|A\right|}-1}.$$

The term $2^{\left|A\right|}-1$ represents the potential cardinality of the proposition *A*, effectively penalizing non-specific evidence. While Shannon entropy collapses when handling the set of all classes (i.e., complete ignorance), $H_{B}\left(m\right)$ reaches its maximum in such states, correctly reflecting high epistemic uncertainty.

This metric serves as the theoretical basis for our fusion strategy, as it allows the system to identify modalities with high information confusion. For cases where $u_{m}\to 1$ (complete ignorance), we set the belief vector to a uniform distribution 1/K and rely on the evidential entropy to down-weight the modality.

### 3.3. Entropy-Aware Evidential Fusion

To enhance the robustness of fusion, we introduce a conflict-aware sequential weighting mechanism. For each modality *m* (except the first in the processing order), we measure conflict with its preceding modality prev(m) by

(10) $$C_{m}\;=\;1-\langle {\tilde{b}}_{m},\;{\tilde{b}}_{\mathrm{prev}(m)}\rangle \in \left[0,1\right],$$

where 〈·,·〉 is the standard inner product on the probability simplex. A smaller $C_{m}$ indicates higher agreement between modalities.

We then accumulate raw modality weights so that both higher uncertainty and higher conflict reduce a modality’s influence:

(11) $$w_{\left(1\right)}=1-u_{\left(1\right)},\;w_{\left(m\right)}=\left(1-u_{\left(m\right)}\right)\left(1-C_{\left(m\right)}\right),\;\;m=2,3,$$

where (1), (2), (3) denote the fixed processing sequence adopted in this work: Text → Audio → Visual. This order is selected based on the generally higher semantic stability of textual features compared to audio-visual signals. In multimodal sentiment analysis and classification tasks, textual data typically exhibits the highest semantic density and lowest aleatoric uncertainty, serving as a robust semantic anchor [39]. Conversely, visual signals often contain significant background noise and redundant information, leading to higher epistemic uncertainty, particularly in long-tailed classes.

The raw modality weights $w_{t}$, $w_{a}$, and $w_{v}$ are first normalized using a temperature-controlled softmax over the set of available modalities M to compute preliminary fusion weights ${\tilde{a}}_{m}$:

(12) $${\tilde{a}}_{m}=\frac{\exp(w_{m}/\eta )}{\sum_{n\in M}\exp\left(w_{n}/\eta \right)},$$

where η is a hyperparameter that controls the sharpness of the weight distribution. A lower η produces a sharper distribution, effectively selecting the single most reliable modality, whereas a higher η encourages a smoother, more democratic integration of multi-source information.

The system then applies an uncertainty-gating mechanism. If the evidential entropy $u_{m}$ exceeds a predefined threshold $\tau_{\mathrm{unc}}$, the weight is adjusted using a decay factor κ:

(13) $${\hat{w}}_{m}={\tilde{a}}_{m}\cdot \left\{\begin{array}{cc} 1, & u_{m}\leq \tau_{\mathrm{unc}},\\ \max\{0.01,\;1-\kappa \left(u_{m}-\tau_{\mathrm{unc}}\right)\}, & u_{m}>\tau_{\mathrm{unc}}. \end{array}\right.$$

After the gating operation, each modality is assigned an adjusted weight ${\hat{w}}_{m}$. An exponential decay function, parameterized by modality uncertainty, is then applied to further normalize ${\hat{w}}_{m}$ and compute the final fusion weight $a_{m}$:

(14) $$a_{m}=\frac{{\hat{w}}_{m}\;\exp\left(-u_{m}\right)}{\sum_{n\in M}{\hat{w}}_{n}\;\exp\left(-u_{n}\right)+\varphi },$$

where $\exp(-u_{m})$ assigns higher weights to modalities with lower uncertainty ($u_{m}$ small), allowing the model to favor more reliable modalities at a fine-grained level. The constant φ serves as a smoothing term to avoid division by zero in the normalization process.

It should be noted that the two normalization steps are complementary rather than conflicting. The first normalization integrates uncertainty and conflict information to produce an initial weight distribution, whereas the second refines this distribution by incorporating gating and exponential decay. This two-step design reflects a trade-off between computational simplicity and fusion effectiveness.

We turn complementarity into action by mapping per-modality evidence and uncertainty into adaptive trust and weights. Low uncertainty and low inter-modal conflict raise a modality’s weight, while the gate suppresses unreliable signals, so reliable and consistent cues dominate the fused evidence, which helps rare classes.

Finally, the fusion coefficient $a_{m}$ is applied to each modality, and a weighted summation is performed over the modality-specific evidence vectors to obtain the final fused evidence vector:

(15) $$e_{\mathrm{final}}=\sum_{m\in M}a_{m}\;e_{m}.$$

Under this uncertainty-driven dynamic fusion scheme, the model can make robust decisions even in the presence of unreliable or conflicting modality information: noisy modalities are suppressed, tail-class information is preserved from being overshadowed, and complementary gains across modalities are effectively exploited. The following section introduces a loss function specifically designed to align with this fusion strategy.

### 3.4. Composite Loss Functions

To ensure equitable contributions across modalities, we propose a composite loss function comprising three components: a Class-Balanced Label-Distribution Aware Margin (CB-LDAM) loss, an EMA-based fairness regularizer, and a cross-modal consistency term.

For a training sample *i* with true label $y_{i}$, let $n_{y_{i}}$ denote the number of training samples in class $y_{i}$. We adopt the LDAM loss [32] to enlarge the decision margins for tail classes. Specifically, the margin reduction for class $y_{i}$ is defined as $\Delta_{y_{i}}$:

(16) $$\Delta_{y_{i}}=\frac{\varpi }{\sqrt[{4}]{n_{y_{i}}}},$$

where ϖ is a hyperparameter of LDAM margin scale. When $n_{y_{i}}$ is small, $\Delta_{y_{i}}$ becomes large, implying that a greater quantity is subtracted from the logit of class $y_{i}$. Consequently, the model must produce a higher raw score for tail classes to be correctly classified, effectively enlarging their decision margins and reducing misclassification caused by class imbalance.

Next, a class frequency weight $\gamma_{y_{i}}$ is introduced for sample *i*, calculated from the frequency of class $y_{i}$ using the formulation of the effective number of samples:

(17) $$\gamma_{y_{i}}=\frac{1-\beta }{1-\beta^{n_{y_{i}}}},\;\beta \in \left[0,1\right),$$

where $n_{y_{i}}$ is the number of training samples in class $y_{i}$, and β∈[0,1) is a hyperparameter. This formulation assigns larger weights to tail classes and smaller weights to head classes, thereby improving generalization across all classes.

Combining the above margin adjustment and class frequency weight, we first construct the margin-adjusted logits:

(18) $$z_{k}\;=\;e_{\mathrm{final},k}\;-\;\Delta_{k}\;1\left[k=y_{i}\right],\;k=1,\ldots ,K,$$

where 1[·] is the indicator function. The posterior probability of the correct class is

(19) $$P\;\left(y_{i}|e_{\mathrm{final}}\;-\;\Delta_{y_{i}}\right)\;=\;\frac{\exp\;\left(z_{y_{i}}\right)}{\sum_{j=1}^{K}\exp\;\left(z_{j}\right)},$$

where $e_{\mathrm{final},k}$ denotes the *k*-th component of the fused evidence vector $e_{\mathrm{final}}$. In this work, we improve the original definition of LDAM, subtracting the class-dependent margin only from the target class to avoid weakening the margin effect that would result from adjusting all logits simultaneously.

Finally, the class-balanced LDAM loss for sample *i* becomes

(20) $$L_{\mathrm{CB}-\mathrm{LDAM},i}\;=\;\gamma_{y_{i}}\;\left[-\log P\;\left(y_{i}|e_{\mathrm{final}}\;-\;\Delta_{y_{i}}\right)\right],$$

where the outer term $\gamma_{y_{i}}$ amplifies the loss contribution of tail class samples, while the inner term logP(·), modified by subtracting the class margin $\Delta_{y_{i}}$, requires the model to have higher confidence for tail classes in order to achieve the same predicted probability. This effectively enlarges the decision boundaries between classes of different frequencies.

Although the above $L_{\mathrm{CB}-\mathrm{LDAM}}$ already balances the loss contributions of different classes to some extent, its weight $\gamma_{y_{i}}$ is statically defined and cannot reflect the real-time variation in class-wise difficulty during training. To address this, we introduce an EMA mechanism to dynamically smooth and track the training loss of each class, thereby automatically adjusting each class’s weight in the total loss. This forms an implicit fairness regularization constraint. For the *k*-th class in training epoch *p*, the EMA of the loss is calculated as:

(21) $${\dot{L}}_{k}^{\left(p\right)}\;=\;\left\{\begin{array}{cc} \psi_{\mathrm{ema}}\;{\dot{L}}_{k}^{\left(p-1\right)}\;+\;\left(1-\psi_{\mathrm{ema}}\right)\;\frac{1}{n_{k}^{\left(b\right)}}\sum_{\;i\in B_{k}}\;L_{\mathrm{CB}-\mathrm{LDAM},i}, & \mathrm{if}\;n_{k}^{\left(b\right)}>0,\\ \psi_{\mathrm{ema}}\;{\dot{L}}_{k}^{\left(p-1\right)}, & \mathrm{if}\;n_{k}^{\left(b\right)}=0, \end{array}\right.$$

where $n_{k}^{\left(b\right)}$ is the number of samples for *k*-th class in the current mini-batch $B_{k}$, and $\psi_{\mathrm{ema}}$ is a smoothing coefficient controlling temporal weighting.

Since the fused evidence is $e_{\mathrm{final}}=\sum_{m}a_{m}e_{m}$, the EMA-derived class weights $\lambda_{\mathrm{fair},k}$ rescale the class-wise gradients that update $e_{m}$. Higher tail evidence lowers $u_{m}=K/\sum_{k}\left(e_{k,m}+1\right)$ and, via the gating $a_{m}\propto \exp\left(-u_{m}\right)$, increases the contribution of reliable modalities to tail classes.

After obtaining the EMA loss ${\dot{L}}_{k}$ for each class, we further compute a fairness weight $\lambda_{\mathrm{fair},k}$ to reallocate the relative contribution of each class in the total loss:

(22) $$\lambda_{\mathrm{fair},k}\;=\;\frac{\exp\;\left(\;\lambda_{\mathrm{fair}}\left({\dot{L}}_{k}-\bar{L}\right)\right)}{\sum_{j=1}^{K}\exp\;\left(\;\lambda_{\mathrm{fair}}\left({\dot{L}}_{j}-\bar{L}\right)\right)},$$

where $\bar{L}=\frac{1}{K}\sum_{j=1}^{K}{\dot{L}}_{j}$ denotes the mean EMA loss across all classes, and $\lambda_{\mathrm{fair}}$ is a sensitivity coefficient controlling how strongly weight discrepancies are amplified based on loss disparities. If the EMA loss of class *k* exceeds the average, it indicates that the model is learning this class less effectively.

Compared to static or manually tuned methods, this EMA-based scheme adaptively adjusts to evolving class-wise performance during training, thereby improving tail-class recognition. Intuitively, instantaneous batch losses are susceptible to noise arising from sampling randomness. The EMA mechanism functions as a temporal low-pass filter, smoothing out these high-frequency fluctuations to reveal the intrinsic learning difficulty of each class. Consequently, this enables the model to dynamically prioritize classes that exhibit persistent under-fitting, effectively shifting the re-weighting strategy from a static count-based prior to a dynamic performance-based adaptation.

To enforce consistent representations across modalities, we introduce a regularization term that penalizes discrepancies between the evidence vectors of different modalities for the same instance:

(23) $$L_{\mathrm{Consistency}}\;=\;\frac{1}{N}\sum_{i=1}^{N}\sum_{m<n}{∥e_{m,i}-e_{n,i}∥}_{2}^{2},$$

where $e_{m,i}$ denotes the evidence vector extracted from modality *m* for sample *i*. This pairwise (no-duplicate) formulation avoids double-counting modality pairs.

The consistency term aligns $e_{k,m}$ for the same sample and class, shrinking the conflict used in prefix weighting (recall $C_{m}=1-\langle {\tilde{b}}_{m},{\tilde{b}}_{\mathrm{prev}(m)}\rangle$). This suppresses spurious inter-modal disagreements and stabilizes the uncertainty-gated weights across modalities.

This loss penalizes the discrepancies between evidence distributions across different modalities for the same sample: the loss remains small only when modalities *m* and *n* produce similar evidence for sample *i*. By minimizing $L_{\mathrm{Consistency}}$, the model is encouraged to reduce cross-modal inconsistency, driving the representations of each modality for the same sample to evolve in a similar discriminative direction. This helps to avoid abnormal embedding bias in a specific modality caused by insufficient data.

Moreover, this alignment implicitly provides a supervisory signal to individual modality branches. By enforcing consistency between $e_{\mathrm{text}}$ and the evidence from other modalities, which eventually forms the strongly supervised $e_{\mathrm{final}}$, $L_{\mathrm{Consistency}}$ ensures the reliability of $e_{\mathrm{text}}$ when used independently for the early exit mechanism during inference.

Fusion turns complementarity into calibrated evidence, and the fairness and consistency terms convert that evidence into larger and cleaner gradients for rare classes, which improves decision margins without extra resampling. During training, the fairness weight amplifies rare class gradients while the consistency term pulls per-modality predictions toward agreement, which stabilizes gating and yields cleaner fused evidence in overlapping classes.

The overall training objective of UMuLT integrates all components as follows:

(1) Classification loss: Compute the CB-LDAM loss $L_{\mathrm{CB}-\mathrm{LDAM},i}$ for all training samples. Update the EMA loss ${\dot{L}}_{k}$ for each class based on current statistics and calculate the corresponding fairness weight $\lambda_{\mathrm{fair},k}$, and apply it to the classification loss of that class.
(2) For each sample *i*, obtain the fairness-weighted classification loss $\lambda_{\mathrm{fair},y_{i}}L_{\mathrm{CB}-\mathrm{LDAM},i}$ using its ground-truth class $y_{i}$.
(3) Consistency regularization: Add the cross-modal consistency term $L_{\mathrm{Consistency}}$, weighted by the balancing coefficient $\delta_{\mathrm{cons}}$, to form the final objective function.

Finally, the total loss for each training batch is:

(24) $$L_{\mathrm{final}}=\sum_{i=1}^{N}\lambda_{\mathrm{fair},y_{i}}L_{\mathrm{CB}-\mathrm{LDAM},i}+\delta_{\mathrm{cons}}L_{\mathrm{Consistency}},$$

where $\delta_{\mathrm{cons}}$ is a predefined regularization coefficient that balances the relative importance between the classification loss and the consistency regularization term.

From the derivation of the composite loss, this design improves overall accuracy while allocating sufficient attention to tail classes and mitigating bias or conflicts during multimodal fusion. In subsequent experiments, the contribution of each module to the final performance is further examined through ablation studies.

The class probabilities are calculated by applying the softmax function to the fused evidence:

(25) $$P\left(y=k|e_{\mathrm{final}}\right)\;=\;\frac{\exp\;\left(e_{\mathrm{final},k}\right)}{\sum_{j=1}^{K}\exp\;\left(e_{\mathrm{final},j}\right)},$$

and the final predicted output:

(26) $$\hat{y}\;=\;\arg\max_{k}\;P\left(y=k|e_{\mathrm{final}}\right).$$

## 4. Experiments

### 4.1. Datasets and Experimental Settings

**MELD** [40]: MELD is widely considered a standard benchmark for multimodal sentiment analysis. It comprises approximately 13,000 conversational samples, with each sample featuring synchronized text, audio, and video streams. The dataset is annotated with seven emotion labels. To investigate the long-tailed distribution problem, we sorted these classes by frequency and categorized them into head, mid, and tail sets. The head group (Neutral and Joy) dominates the dataset, constituting about 63.8% of the training data. The mid group (Surprise and Anger) accounts for 23.7%, while the tail group (Sadness, Disgust, and Fear) represents a scarce 12.5%. We adhere to the standard data split: 9989 samples for training, 1109 for validation, and 2610 for testing.

**Charades** [41]: Charades is a large-scale dataset focused on indoor activities, containing 9848 videos equipped with synchronized text, audio, and visual data. It is notable for its dense annotations, which include free-form descriptions and over 66,500 action intervals spanning 157 classes. For our long-tailed experiments, we ranked the 157 classes by instance count and divided them into three segments: 100 head classes, 37 mid classes, and 20 tail classes.

**COIN** [42]: COIN is a comprehensive instructional video dataset covering 180 tasks across 12 distinct domains. It contains a total of 11,827 videos, amounting to approximately 476 h of footage. It provides 46,354 temporally annotated step segments, with an average of 3.91 steps per video and 14.91 s per step. We adopt the official split with 9030 training videos and 2797 test videos.

We report performance by partitioning classes on the training split by per-class instance counts: head (>100), mid (20–100), and tail (<20). Counts are measured as utterances for MELD, action intervals for Charades, and step segments for COIN.

**Implementation Details.** All experiments were implemented in Python 3.12.7, conducted on a workstation equipped with an NVIDIA RTX 5090D GPU, an AMD 9950X CPU, and 96 GB of RAM. We systematically tuned the training core hyperparameters to boost the model’s performance. Table 1 shows the final hyperparameters:

### 4.2. Evaluation Metrics

In this section, we evaluate performance on the MELD, Charades, and COIN datasets using five metrics: accuracy (ACC), F1-score (F1), geometric mean (G-mean), area under the ROC curve (AUC), and expected calibration error (ECE). Each metric is reported both as a macro average across all classes and as separate averages for the head, mid, and tail subsets, thereby providing a comprehensive evaluation under a long-tailed distribution.

For each class *i*, we compute the evaluation metrics using the standard confusion matrix $\left(TP_{i},\;FP_{i},\;TN_{i},\;FN_{i}\right)$:

(27) $$ACC_{i}=\frac{TP_{i}+TN_{i}}{TP_{i}+FP_{i}+TN_{i}+FN_{i}},$$

(28) $$F1_{i}=\frac{2TP_{i}}{2TP_{i}+FP_{i}+FN_{i}},$$

(29) $$G-mean_{i}=\sqrt{\frac{TP_{i}}{TP_{i}+FN_{i}}\cdot \frac{TN_{i}}{TN_{i}+FP_{i}}},$$

(30) $$ECE_{i}=\sum_{b=1}^{B}\frac{|b_{i}|}{N_{i}}\left|ACC_{i}\left(b\right)-conf_{i}\left(b\right)\right|,$$

where *B* is the index of confidence bins, $|b_{i}|$ and $N_{i}$ denote the number of samples in bin *b* and the total number of samples in class *i*, respectively, while $ACC_{i}\left(b\right)$ and $conf_{i}\left(b\right)$ represent the empirical accuracy and mean confidence in bin *b*. Additionally, $AUC_{i}$ refers to the area under the ROC curve for class *i*.

The overall macro metrics are calculated as the average over all classes:

(31) $$\Phi_{\mathrm{Macro}}=\frac{1}{C}\sum_{i=1}^{C}\Phi_{i},$$

where *C* is the number of classes.

For the head, mid, and tail subsets, the macro-averaged metrics are computed as follows:

(32) $$\Phi_{\mathrm{Head}}=\frac{1}{N_{\mathrm{Head}}}\sum_{i\in E_{\mathrm{Head}}}\Phi_{i},$$

(33) $$\Phi_{\mathrm{Mid}}=\frac{1}{N_{\mathrm{Mid}}}\sum_{i\in E_{\mathrm{Mid}}}\Phi_{i},$$

(34) $$\Phi_{\mathrm{Tail}}=\frac{1}{N_{\mathrm{Tail}}}\sum_{i\in E_{\mathrm{Tail}}}\Phi_{i},$$

where Φ∈{ACC, F1, G-mean, AUC, ECE}, and $E_{\mathrm{Head}},\;E_{\mathrm{Mid}},\;E_{\mathrm{Tail}}$ denote the indices of the head, mid, and tail classes, respectively, with corresponding sample sizes $N_{\mathrm{Head}},\;N_{\mathrm{Mid}},\;N_{\mathrm{Tail}}$. For tail classes $E_{\mathrm{Tail}}$ and per-class scores $\left\{\Phi_{i}\right\}$, where Φ∈{ACC, F1, G-mean, AUC, ECE}:

Higher-is-better (ACC/F1/G-mean/AUC):

$$\mathrm{WorstTail}=\min_{i\in E_{\mathrm{Tail}}}\Phi_{i},\;P25\mathrm{Tail}\left(\Phi \right)={\mathrm{Quantile}}_{0.25}\left\{\Phi_{i}:i\in E_{\mathrm{Tail}}\right\}.$$

Lower-is-better (ECE):

$$\mathrm{WorstTail}\left(\mathrm{ECE}\right)=\max_{i\in E_{\mathrm{Tail}}}{\mathrm{ECE}}_{i},\;P25\mathrm{Tail}\left(\mathrm{ECE}\right)={\mathrm{Quantile}}_{0.75}\left\{{\mathrm{ECE}}_{i}:i\in E_{\mathrm{Tail}}\right\},$$

where ${\mathrm{Quantile}}_{\alpha }$ refers to the empirical α-quantile over the tail classes.

### 4.3. Comparisons with Other Methods

To demonstrate the effectiveness of our proposed approach, eight long-tailed classification methods used for comparison are as follows:

Unimodal long-tailed baselines:

- **TLC** [18]: A trustworthy learning scheme for long-tailed classification that combines re-weighting/re-sampling with uncertainty-aware training and calibration to curb head overconfidence and improve tail reliability.
- **CBERL** [2]: Class-boundary enhanced representation learning that enlarges inter-class margins and tightens intra-class clusters (via margin losses and neighbor-aware contrast), strengthening tail discriminability.
- **BBN** [43]: A bilateral-branch network with a conventional and a re-balanced branch sharing early layers; a curriculum-style aggregation progressively boosts tail classes.
- **FFN** [33]: A multi-branch feature-fusion framework that trains on natural and re-balanced distributions and adaptively fuses branch outputs at inference to balance head–tail performance.

Multimodal long-tailed baselines:

- **VL-LTR** [44]: Builds class-wise vision–language prototypes and optimizes with re-balanced objectives to improve tail separability and generalization.
- **BALLAD** [16]: A strong yet simple VL baseline that continues contrastive pretraining and uses lightweight adapters to transfer pretrained VL models to long-tailed regimes with minimal changes.
- **MMoE** [45]: Multi-gate Mixture-of-Experts, a classic multi-task learning architecture that employs a gating network to dynamically select and weight expert networks based on the input. Each expert processes the shared multimodal features independently, and the gating network determines the contribution of each expert to the final prediction.
- **Candle** [46]: Efficient long-tailed adaptation for pretrained VL models, combining compensating logit adjustment (CLA), cross-modal attention, and virtual prototypes to deliver consistent tail gains at low cost.

For a fair comparison, all baselines use the same frozen text/audio/visual encoders as UMuLT. For VL-LTR, BALLAD, and Candle (originally vision–language), we adapt them to the tri-modal setting by concatenating text–audio–visual embeddings and training their official classifier heads with their published objectives. TLC treats each modality as an evidential expert; we apply its uncertainty-weighted fusion rule to combine predictions from the three modality-specific experts using their associated uncertainty estimates. The remaining components are kept consistent with the original implementation. FFN fuses concatenated features through two parallel MLP branches, whose outputs are combined by a learnable coefficient, following its original early-fusion formulation. MMoE uses three modality-specific expert networks with a shared gating mechanism over concatenated features, trained with our loss. BBN duplicates the fused feature stream into “main” and “re-balanced” branches, interpolating their outputs via the scheduling function used in the original design.

**Overall Performance Comparison.** The experimental results are summarized in Table 2. On MELD, UMuLT achieves the highest head and tail scores while trailing FFN on the mid split, yielding a more balanced head–tail trade-off without degrading head performance. On Charades, UMuLT leads in overall and head metrics, whereas FFN and TLC obtain the best mid and tail results, respectively. On COIN, UMuLT delivers the best overall and tail performance and the best mid F1/G-mean, while Candle (head ACC/G-mean) and CBERL (mid ACC) are competitive on specific splits. The best values are marked in bold in the experimental results.

Overall, the proposed evidence-based uncertainty-gated fusion and fairness regularization consistently improve both overall accuracy and tail class recall.

### 4.4. Comparison with Simple Fusion Baseline

To validate that UMuLT’s improvement stems from its uncertainty-aware fusion mechanism rather than simply combining multiple modalities, we conducted additional experiments comparing against a fundamental baseline: Late Fusion with Average Softmax. This baseline independently processes each modality through its respective backbone (Text-BERT, Audio-ResNet18, Visual-ResNet50) and then averages the predicted probability distributions:

(35) $$p_{\mathrm{avg}}=\frac{1}{3}\left(p_{\mathrm{text}}+p_{\mathrm{audio}}+p_{\mathrm{visual}}\right),$$

where $p_{\mathrm{text}}$, $p_{\mathrm{audio}}$, and $p_{\mathrm{visual}}$ are the softmax outputs from the three unimodal models, respectively. The final prediction is $\hat{y}=\arg\max\left(p_{\mathrm{avg}}\right)$.

Table 3 presents the comprehensive comparison between the Average Softmax baseline and UMuLT across all three datasets. The results demonstrate that while simple averaging can aggregate multimodal information, it significantly underperforms UMuLT’s evidence-based fusion mechanism.

UMuLT achieves higher overall accuracy compared to simple averaging, demonstrating that uncertainty-aware fusion significantly outperforms naive probability aggregation. The performance gap is particularly pronounced on tail classes. This validates that uncertainty quantification is crucial for handling rare categories.

### 4.5. Calibration Study

**Figure 3:**
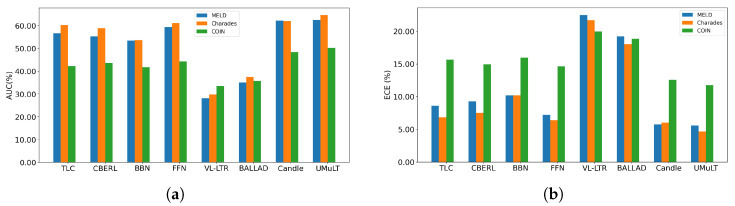
Performance comparison: (**a**) AUC and (**b**) ECE.

Figure 3 complements the preceding accuracy-oriented results by reporting the AUC and the ECE. As shown in Figure 3a, UMuLT attains the highest AUC on MELD, Charades, and COIN, while Candle is a close second across all datasets. In Figure 3b, UMuLT achieves the lowest ECE on all three datasets, with statistically significant improvements on Charades (*p* = 0.006) and COIN (*p* = 0.010), while the MELD improvement is numerically small and not statistically significant.

To complement macro averages, we summarize tail behavior with two F1-based statistics. WorstTail is the minimum class-wise F1 over the tail set, $\min_{c\in \mathrm{Tail}}F1_{c}$. P25Tail is the 25th percentile of ${\left\{F1_{c}\right\}}_{c\in \mathrm{Tail}}$. Both are computed on tail classes defined by the training split. We compare UMuLT with Baseline-X in Table 4, the strongest and most stable non-UMuLT baseline across datasets; Baseline-X denotes Candle.

Overall, combining near-top AUC with consistently lower ECE, UMuLT yields more reliable and better-calibrated predictions while improving the worst and lower-quartile tail classes compared with state-of-the-art baselines such as Candle.

### 4.6. Visualization Study

To understand how evidence-based, uncertainty-gated fusion behaves on head and tail examples, we visualize per-modality evidence and predictive uncertainty.

**Figure 4:**
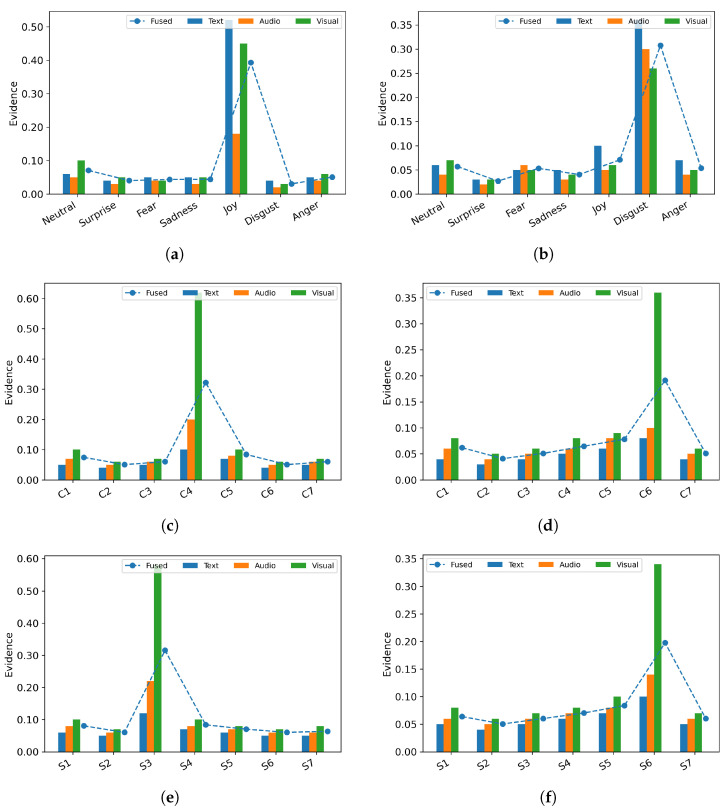
Evidence distribution for head and tail class samples across different datasets. (**a**) Head class samples on MELD. (**b**) Tail class samples on MELD. (**c**) Head class samples on Charades. (**d**) Tail class samples on Charades. (**e**) Head class samples on COIN. (**f**) Tail class samples on COIN.

Figure 4 plots per-modality evidence for text, audio, and visual together with the fused evidence on representative head and tail samples: For MELD, each position on the x-axis corresponds to one of the seven emotion classes; for Charades, classes are labeled C1–C7; for COIN, classes are labeled S1–S7. Across all three datasets, when a unimodal shows a pronounced spike, for example Charades head C4 and tail C6, the fused curve rises accordingly. This behavior mirrors a human expert’s intuition: for instance, in a video where audio cues are occluded but the visual instruction is clear, the system correctly places trust in the visual stream. This indicates that the fusion mechanism preserves strong, reliable cues while filtering out noise, acting as an adaptive decision filter.

**Figure 5:**
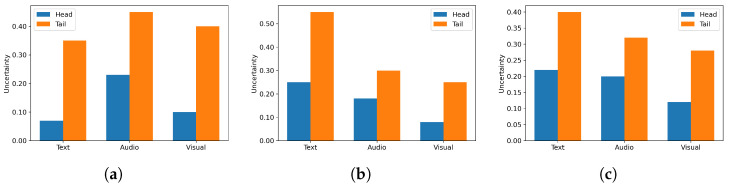
Uncertainty comparison between head and tail samples. (**a**) Comparison on MELD. (**b**) Comparison on Charades. (**c**) Comparison on COIN.

Figure 5a–c report per-modality uncertainty on head and tail samples. Tail samples show higher uncertainty across all modalities. The largest head–tail gap is dataset dependent: Text on MELD and Charades, Visual on COIN, with Audio the smallest throughout. These results support our design: UMuLT gates weak modalities while passing through strong, well-evidenced signals, reducing ambiguity on tail cases.

### 4.7. Ablation Study

We conduct two ablations to quantify the contribution of modalities and modules. Table 5 and Table 6 report ACC, F1, and G-mean on the Head/Mid/Tail splits.

**Modality Ablation.** Across datasets, fusing Text and Visual is the strongest two-modality setting, indicating complementary linguistic–visual cues; Audio adds further gains in the full tri-modal model. The full model achieves the best All/Head/Mid/Tail scores on every dataset.

**Module Ablation.** Removing cross-modal alignment or the uncertainty gate causes the largest drops—especially on G-mean and tail, showing that aligning and filtering weak modalities is critical for robustness. Disabling the class-balanced LDAM loss also degrades performance under skew. EMA-based fairness tracking and consistency regularization yield additional, consistent gains. The full model performs best, confirming the modules’ synergy under long-tailed multimodal learning.

These results confirm that simply throwing multiple modalities at the problem isn’t enough; we need specific architectural choices to handle the long-tailed distribution. The drop in performance on MELD is particularly telling. It shows that the cross-modal alignment and the uncertainty gate are not just independent modules—they depend on each other. If we have alignment but no gate, the model just spreads noise around. On the flip side, if we try to gate without alignment, the features are too disconnected for the system to accurately spot conflicts. This mutual dependency confirms that effective evidential filtering relies on coherent, aligned representations.

### 4.8. Sensitivity of Hyper-Parameters Study

We study three hyper-parameters on MELD: the consistency weight $\delta_{\mathrm{cons}}$, the fairness coefficient $\lambda_{\mathrm{fair}}$, and the uncertainty gate threshold $\tau_{\mathrm{unc}}$. All sensitivity curves use the validation split with a reduced training budget, so absolute accuracies are lower than the final Full results in Table 2; we focus on relative trends.

**Figure 6:**
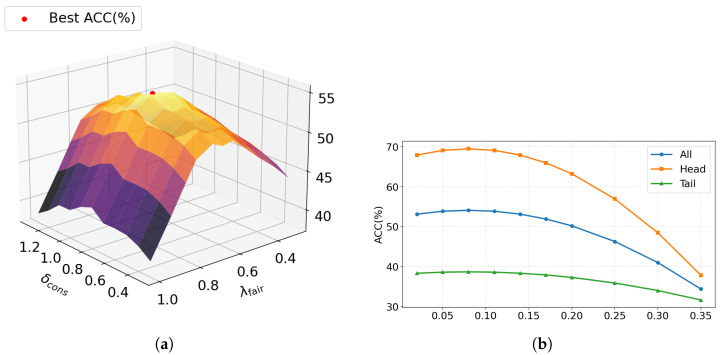
Sensitivity of hyper-parameters. (**a**) Sensitivity of hyper-parameters of loss function on MELD. (**b**) Sensitivity of hyper-parameters of uncertainty gate threshold on MELD.

Figure 6a sweeps the pair $\delta_{\mathrm{cons}},\;\lambda_{\mathrm{fair}}$ on MELD. Accuracy follows a convex pattern: increasing either term helps up to a point, then saturates or declines, showing complementary effects. The optimum is near $\delta_{\mathrm{cons}}=0.80$ and $\lambda_{\mathrm{fair}}=0.65$, which we adopt for MELD and Charades; for COIN we keep $\lambda_{\mathrm{fair}}=0.65$ and use $\delta_{\mathrm{cons}}=0.70$ given its label distribution, see Table 1.

Figure 6b sweeps $\tau_{\mathrm{unc}}$ on MELD and reports All, Head, and Tail accuracies. Performance is bell-shaped with a broad optimum between 0.08 and 0.12: small thresholds retain noisy modalities and hurt tail, while large thresholds drop informative signals and hurt head. We therefore set $\tau_{\mathrm{unc}}=0.08$ for MELD and Charades and 0.09 for COIN, see Table 1.

### 4.9. Significance Testing

We assess statistical significance with a two-sided paired bootstrap over test instances. We compare UMuLT with Candle in Table 7. For each metric Φ we compute the paired difference $\Delta =\Phi_{\mathrm{Ours}}-\Phi_{\mathrm{Candle}}$ and report the bootstrap *p*-value and the Benjamini–Hochberg false discovery rate adjusted *q* for each dataset. Bold p-values indicate statistical significance at the 0.05 level.

### 4.10. Computational Complexity Analysis

UMuLT supports two inference modes: (1) Full mode: all three modalities are processed and fused via the uncertainty-gated mechanism. This mode is used for samples near decision boundaries or when text-only confidence falls below the early-exit threshold. (2) Dynamic mode: high-confidence samples bypass the audio and visual backbones through the text-only early-exit mechanism, significantly reducing computational cost. Table 8 presents the detailed complexity comparison.

## 5. Discussion

Based on the experimental results from the MELD, Charades, and COIN datasets, the proposed UMuLT framework demonstrates robust performance under long-tailed distributions. Specifically, UMuLT surpasses baselines in overall accuracy (ACC) and achieves competitive or superior tail-class accuracy (Tail-ACC) across datasets. Moreover, UMuLT consistently gets the lowest ECE scores across all datasets. This proves that adding Belief Entropy really helps to make the predictions more reliable. The ablation results clearly show that both the uncertainty gate and cross-modal alignment are essential. If we remove either one, the performance—especially on tail classes—takes a notable hit. These findings confirm that our framework effectively filters out noisy modalities and forces the model to learn better features for rare categories.

We believe the key reason for this success is that we strictly separate aleatoric uncertainty from epistemic uncertainty. Aleatoric uncertainty is just inherent noise (like background sounds), whereas epistemic uncertainty comes from a lack of knowledge due to data scarcity. Most standard methods tend to blur the line between these two, leading to overconfidence on rare samples. Our Belief Entropy mechanism, however, specifically targets the epistemic part. It measures the model’s “ignorance” and acts as a safety valve. Instead of merely suppressing noise, it stops the system from making high-confidence guesses when the evidence is insufficient, thus tackling the core issue of long-tailed recognition.

It is worth noting that while UMuLT wins on Head and Tail subsets, it lags slightly behind FFN on the MELD mid split. This is a trade-off. FFN keeps some ambiguous features that might help with overlapping intermediate classes, whereas UMuLT actively filters out high-uncertainty signals to prevent noise propagation. This leads to a more conservative strategy on ambiguous samples. Despite the demonstrated robustness, we acknowledge potential limitations of the proposed framework. The incorporation of evidential reasoning and cross-modal consistency introduces additional computational complexity, resulting in higher training costs compared to deterministic baselines. Furthermore, the performance advantage of UMuLT may diminish in scenarios characterized by perfectly balanced distributions or high-fidelity data, where the heavy reliance on uncertainty quantification becomes less critical. Future investigations will address these constraints by optimizing training efficiency and exploring adaptive mechanisms for dynamic modality availability.

## 6. Conclusions

We presented UMuLT, a robust hybrid intelligent framework for long-tailed multimodal classification that effectively integrates evidence-based uncertainty reasoning, EMA-driven fairness, and cross-modal consistency within a two-stage optimization process. By bridging the gap between deep representation learning and evidential decision-making, the three components act synergistically: the gate suppresses unreliable modalities, fairness tracking rebalances learning toward the tail, and consistency sharpens boundaries under modality discrepancy. Across three different datasets, UMuLT consistently outperforms competing methods, delivering higher overall performance, improved calibration, and stronger tail-class robustness, with gains validated by bootstrap significance tests. In future work, we will study efficiency and stability under varied deployment conditions; specifically, we plan to develop adaptive consistency schedules and extend uncertainty-gated fusion to handle dynamically missing or newly added modalities, while reducing computational complexity.

## Figures and Tables

**Table 1:** Experimental hyperparameter settings.

Hyperparameter	MELD	Charades	COIN
Learning rate $\eta_{l}$	$2\;\times \;10^{-5}$	$2\;\times \;10^{-5}$	$2\;\times \;10^{-5}$
Batch size	64	64	64
Early-stop patience	6	6	6
Max epochs	120	140	120
EMA smoothing $\psi_{\mathrm{ema}}$	0.995	0.995	0.994
Consistency weight $\delta_{\mathrm{cons}}$	0.80	0.80	0.70
Uncertainty decay κ	0.85	0.88	0.92
Uncertainty gate threshold $\tau_{\mathrm{unc}}$	0.08	0.08	0.09
Fairness sensitivity coeff. $\lambda_{\mathrm{fair}}$	0.65	0.65	0.65
LDAM margin scale ϖ	1.00	0.50	0.65
Class-balanced β	0.9997	0.9985	0.9988

**Table 2:** Classification performance comparison on datasets.

Dataset	Model	ACC				F1				G-Mean			
		All	Head	Mid	Tail	All	Head	Mid	Tail	All	Head	Mid	Tail
MELD	TLC	56.55	62.69	48.83	28.15	49.86	56.42	41.51	25.01	41.52	47.02	34.18	26.35
	CBERL	55.21	61.11	49.50	34.84	48.69	55.00	42.08	27.87	40.54	45.83	34.65	23.69
	BBN	53.38	59.67	46.46	33.23	47.10	53.70	39.49	26.58	39.21	44.75	32.52	22.60
	FFN	59.28	65.30	**54.50**	36.50	52.27	58.77	**46.33**	29.21	43.51	48.97	**38.15**	24.82
	VL-LTR	28.07	30.61	26.01	18.59	24.74	27.54	22.10	14.87	20.59	22.95	18.20	12.64
	BALLAD	34.95	39.08	30.41	21.70	30.84	35.17	25.85	17.36	25.67	29.31	21.29	14.76
	MMoE	58.73	64.81	51.31	37.22	51.84	58.32	43.60	29.76	43.15	48.56	35.89	25.28
	Candle	62.16	69.48	54.12	38.68	54.84	62.53	46.00	**30.94**	45.66	52.11	37.88	26.30
	UMuLT	**62.50**	**70.71**	52.00	**39.01**	**55.17**	**63.63**	44.20	28.42	**45.93**	**53.03**	36.40	**26.52**
Charades	TLC	60.25	63.22	49.04	**38.75**	53.21	56.81	41.97	**29.34**	44.91	45.68	34.46	**25.56**
	CBERL	58.89	61.41	45.11	24.81	52.59	55.21	41.19	21.18	44.70	46.93	35.66	19.85
	BBN	53.59	56.48	40.02	28.52	47.89	50.84	34.02	22.82	39.88	42.36	28.01	19.40
	FFN	61.09	63.04	**51.53**	31.97	54.54	56.74	**42.74**	25.58	45.39	47.28	**35.82**	21.74
	VL-LTR	31.11	32.88	27.01	19.41	27.96	28.70	23.11	15.22	22.34	24.00	19.10	13.21
	BALLAD	36.91	38.71	31.00	22.32	32.81	34.98	26.20	18.00	27.16	28.91	21.55	15.12
	MMoE	60.47	63.58	50.12	35.60	53.92	57.20	42.65	28.48	45.23	47.65	35.08	24.19
	Candle	61.90	65.09	46.52	36.52	55.31	58.58	39.55	29.21	46.06	48.82	32.57	24.83
	UMuLT	**64.54**	**68.01**	47.97	36.05	**57.68**	**61.21**	40.78	28.84	**48.03**	**51.01**	33.58	24.51
COIN	TLC	42.28	52.04	38.53	28.35	36.78	46.83	32.75	22.68	30.61	39.03	26.97	19.28
	CBERL	43.59	50.09	**45.76**	26.20	36.67	42.58	38.60	20.96	31.24	37.57	31.33	17.81
	BBN	41.68	53.07	35.78	28.02	36.00	47.11	30.41	22.42	30.21	39.80	25.05	19.05
	FFN	44.22	56.09	38.14	29.90	37.28	47.68	32.42	23.92	32.05	42.07	26.70	20.33
	VL-LTR	33.41	42.13	29.24	22.36	28.17	35.81	24.86	17.89	24.21	31.60	20.47	15.21
	BALLAD	35.68	44.02	32.48	23.75	30.08	37.41	27.60	19.00	25.83	33.01	22.73	16.15
	MMoE	46.82	58.45	41.08	30.25	39.52	49.68	34.92	24.18	33.95	43.48	28.77	20.54
	Candle	48.32	**63.25**	40.49	30.60	40.75	53.76	34.42	24.48	35.05	**47.43**	28.34	20.71
	UMuLT	**50.20**	62.06	45.47	**33.53**	**43.62**	**55.75**	**38.65**	**26.83**	**36.35**	46.54	**31.83**	**22.80**

**Table 3:** Comparison between Average Softmax baseline and UMuLT across datasets.

Dataset	Model	ACC				F1				G-Mean			
		All	Head	Mid	Tail	All	Head	Mid	Tail	All	Head	Mid	Tail
MELD	Avg-Softmax	55.20	60.85	49.19	34.51	48.65	55.11	43.34	27.61	40.50	45.90	33.23	23.25
	UMuLT	**62.50**	**70.71**	**52.00**	**39.01**	**55.17**	**63.63**	**44.20**	**28.42**	**45.93**	**53.03**	**36.40**	**26.52**
Charades	Avg-Softmax	55.40	58.64	45.81	34.21	49.35	52.75	38.90	27.35	41.53	44.30	32.01	23.16
	UMuLT	**64.54**	**68.01**	**47.97**	**36.05**	**57.68**	**61.21**	**40.78**	**28.84**	**48.03**	**51.01**	**33.58**	**24.51**
COIN	Avg-Softmax	42.65	53.22	37.15	26.41	35.85	45.20	31.61	21.15	30.95	39.95	26.01	18.11
	UMuLT	**50.20**	**62.06**	**45.47**	**33.53**	**43.62**	**55.75**	**38.65**	**26.83**	**36.35**	**46.54**	**31.83**	**22.80**

**Table 4:** Tail-focused snapshot on tail classes.

Dataset	Method	WorstTail (F1)	P25Tail (F1)
MELD	Candle	20.4	24.6
	Ours	**22.1**	**26.0**
Charades	Candle	15.3	22.8
	Ours	**17.1**	**24.0**
COIN	Candle	12.0	18.5
	Ours	**14.5**	**20.9**

**Table 5:** Modality ablation on datasets.

Dataset	Modality	ACC				F1				G-Mean			
		All	Head	Mid	Tail	All	Head	Mid	Tail	All	Head	Mid	Tail
MELD	Text	48.99	63.21	48.63	35.12	42.11	56.89	41.33	28.09	35.11	47.41	34.04	23.88
	Audio	47.66	61.50	47.31	34.17	40.96	55.35	40.21	27.33	34.16	46.12	33.11	23.23
	Visual	52.04	67.42	51.86	36.84	44.75	60.68	44.08	29.47	37.31	50.57	36.30	25.05
	Text + Audio	55.80	64.05	49.15	35.60	48.70	57.30	41.70	28.50	41.30	47.80	34.50	24.20
	Audio + Visual	56.90	66.50	51.40	36.20	49.70	59.10	43.90	28.90	42.00	49.60	35.60	24.80
	Text + Visual	58.90	68.00	51.70	37.10	51.30	60.90	43.80	29.70	43.20	50.90	36.10	25.20
	Full	**62.50**	**70.71**	**52.00**	**39.01**	**55.17**	**63.63**	**44.20**	**28.42**	**45.93**	**53.03**	**36.40**	**26.52**
Charades	Text	51.67	59.37	42.58	30.85	45.00	53.37	35.94	24.77	37.92	44.82	29.71	21.04
	Audio	50.35	57.66	41.20	30.03	43.86	52.29	35.06	24.08	36.89	43.60	28.99	20.40
	Visual	54.85	63.11	45.07	32.52	48.07	56.84	38.40	26.09	40.40	47.83	32.03	22.37
	Text + Audio	55.76	59.80	43.30	31.42	49.18	54.33	36.49	25.30	41.82	45.40	30.47	21.34
	Audio + Visual	58.07	63.49	45.84	32.72	51.61	57.37	38.81	26.16	43.48	47.96	31.84	22.31
	Text + Visual	60.49	65.44	46.67	33.46	53.66	58.99	39.37	26.81	45.35	49.79	32.82	22.74
	Full	**64.54**	**68.01**	**47.97**	**36.05**	**57.68**	**61.21**	**40.78**	**28.84**	**48.03**	**51.01**	**33.58**	**24.51**
COIN	Text	40.95	52.80	39.60	28.50	34.80	47.80	33.50	22.90	29.40	40.20	27.80	19.40
	Audio	39.70	51.70	38.40	27.90	33.90	46.90	32.60	22.40	28.60	39.40	27.10	18.90
	Visual	43.40	56.10	41.90	30.20	37.10	50.90	35.60	24.40	31.60	42.80	29.90	20.90
	Text + Audio	42.30	53.50	40.50	29.20	36.30	48.90	34.20	23.50	30.90	40.90	28.70	19.80
	Audio + Visual	44.70	57.80	43.20	30.90	38.60	52.40	36.80	24.70	32.80	44.10	30.40	21.10
	Text + Visual	46.90	59.90	44.50	31.70	40.70	54.00	37.70	25.30	34.60	45.80	31.50	21.60
	Full	**50.20**	**62.06**	**45.47**	**33.53**	**43.62**	**55.75**	**38.65**	**26.83**	**36.35**	**46.54**	**31.83**	**22.80**

**Table 6:** Module ablation on datasets.

Dataset	Setting	ACC				F1				G-Mean			
		All	Head	Mid	Tail	All	Head	Mid	Tail	All	Head	Mid	Tail
MELD	w/o Cross-Align.	58.05	65.11	50.05	35.90	51.23	58.60	42.55	28.72	42.65	48.83	35.04	24.41
	w/o Uncert. Gate	58.02	65.07	50.02	35.88	51.20	58.56	42.52	28.71	42.63	48.80	35.02	24.40
	w/o CB-LDAM	58.99	66.16	50.86	36.48	52.06	59.55	43.23	29.19	43.34	49.62	35.60	24.81
	w/o EMA Fairness	59.39	66.61	51.21	36.73	52.41	59.95	43.52	29.38	43.63	49.96	35.84	24.98
	w/o Consistency Reg.	59.93	67.22	51.67	37.07	52.89	60.49	43.92	29.65	44.03	50.41	36.17	25.20
	Full	**62.50**	**70.71**	**52.00**	**39.01**	**55.17**	**63.63**	**44.20**	**28.42**	**45.93**	**53.03**	**36.40**	**26.52**
Charades	w/o Cross-Align.	59.43	64.56	43.97	32.72	53.05	57.99	37.24	26.21	44.19	48.29	30.82	22.32
	w/o Uncert. Gate	59.08	64.04	44.26	32.21	52.83	57.53	37.67	25.72	43.76	47.69	31.02	21.92
	w/o CB-LDAM	60.58	65.50	44.89	33.22	54.23	58.77	38.31	26.49	45.07	48.97	31.55	22.63
	w/o EMA Fairness	61.47	65.81	45.35	33.53	54.81	59.45	38.74	26.78	45.52	49.45	31.90	22.77
	w/o Consistency Reg.	62.27	66.47	46.26	34.18	55.57	59.79	39.31	27.37	46.28	49.73	32.30	23.33
	Full	**64.54**	**68.01**	**47.97**	**36.05**	**57.68**	**61.21**	**40.78**	**28.84**	**48.03**	**51.01**	**33.58**	**24.51**
COIN	w/o Cross-Align.	47.62	59.30	43.60	31.40	41.08	52.90	36.80	25.10	34.00	44.30	29.90	21.30
	w/o Uncert. Gate	47.48	59.14	43.45	31.32	40.96	52.76	36.70	25.02	33.89	44.17	29.82	21.23
	w/o CB-LDAM	48.26	60.02	43.28	31.88	41.63	53.56	37.35	25.49	34.46	44.86	30.30	21.60
	w/o EMA Fairness	48.62	60.45	44.56	32.15	41.96	53.95	37.64	25.70	34.74	45.19	30.53	21.79
	w/o Consistency Reg.	49.14	61.00	45.00	32.50	42.41	54.47	38.02	26.00	35.13	45.65	30.86	22.05
	Full	**50.20**	**62.06**	**45.47**	**33.53**	**43.62**	**55.75**	**38.65**	**26.83**	**36.35**	**46.54**	**31.83**	**22.80**

**Table 7:** Significance testing results of improvements using a two-sided paired bootstrap.

Dataset	Endpoint	Δ	$p_{\mathrm{boot}}$ (*q*)
MELD	F1_Macro	+0.009	**0.012** (0.024)
	P25Tail (F1)	+0.016	**0.002** (0.008)
	ECE_Macro	−0.002	0.180 (0.180)
	P25Tail (ECE)	−0.010	**0.020** (0.027)
Charades	F1_Macro	+0.007	**0.021** (0.021)
	P25Tail (F1)	+0.013	**0.009** (0.012)
	ECE_Macro	−0.013	**0.006** (0.012)
	P25Tail (ECE)	−0.018	**0.004** (0.012)
COIN	F1_Macro	+0.029	**0.001** (0.002)
	P25Tail (F1)	+0.024	**0.001** (0.002)
	ECE_Macro	−0.009	**0.010** (0.010)
	P25Tail (ECE)	−0.012	**0.008** (0.010)

**Table 8:** Computational complexity comparison under different inference modes.

Dataset	Mode	FLOPs (G)	Time (ms)
MELD	Backbones Only	12.41	42
	UMuLT (Full)	13.10	45
	UMuLT (Dynamic)	7.82	28
Charades	Backbones Only	15.87	51
	UMuLT (Full)	16.75	54
	UMuLT (Dynamic)	9.24	31
COIN	Backbones Only	13.95	46
	UMuLT (Full)	14.74	49
	UMuLT (Dynamic)	8.67	30

## Data Availability

The datasets and code used in this study are available from the author on reasonable request and will be released in a public repository upon publication.
